# Sensitive Quantitative Analysis of the Meconium Bacterial Microbiota in Healthy Term Infants Born Vaginally or by Cesarean Section

**DOI:** 10.3389/fmicb.2016.01997

**Published:** 2016-12-15

**Authors:** Ravinder Nagpal, Hirokazu Tsuji, Takuya Takahashi, Kazunari Kawashima, Satoru Nagata, Koji Nomoto, Yuichiro Yamashiro

**Affiliations:** ^1^Probiotics Research Laboratory, Graduate School of Medicine, Juntendo UniversityTokyo, Japan; ^2^Yakult Central InstituteTokyo, Japan; ^3^Gonohashi Obstetrics and Gynecology HospitalTokyo, Japan; ^4^Department of Pediatrics, School of Medicine, Tokyo Women’s Medical UniversityTokyo, Japan

**Keywords:** C-section, dysbiosis, gut bacteria, intestinal microbiota, *Lactobacillus*, meconium, RT-qPCR

## Abstract

For decades, babies were thought to be born germ-free, but recent evidences suggest that they are already exposed to various bacteria *in utero*. However, the data on population levels of such pioneer gut bacteria, particularly in context to birth mode, is sparse. We herein aimed to quantify such bacteria from the meconium of 151 healthy term Japanese infants born vaginally or by C-section. Neonatal first meconium was obtained within 24–48 h of delivery; RNA was extracted and subjected to reverse-transcription-quantitative PCR using specific primers for *Clostridium coccoides* group, *C. leptum* subgroup, *Bacteroides fragilis* group, *Atopobium* cluster, *Prevotella, Bifidobacterium*, *Lactobacillus*, *Enterococcus*, Enterobacteriaceae, *Staphylococcus*, *Enterococcus*, *Streptococcus*, *C. perfringens*, and *C. difficile*. We detected several bacterial groups in both vaginally- and cesarean-born infants. *B. fragilis* group, Enterobacteriaceae, *Enterococcus*, *Streptococcus*, and *Staphylococcus* were detected in more than 50% of infants, with counts ranging from 10^5^ to 10^8^ cells/g sample. About 30–35% samples harbored *Bifidobacterium* and *Lactobacillus* (10^4^–10^5^ cells/g); whereas *C. coccoides* group, *C. leptum* subgroup and *C. perfringens* were detected in 10–20% infants (10^3^–10^5^ cells/g). Compared to vaginally-born babies, cesarean-born babies were significantly less often colonized with *Lactobacillus* genus (6% vs. 37%; *P* = 0.01) and *Lactobacillus gasseri* subgroup (6% vs. 31%; *P* = 0.04). Overall, seven *Lactobacillus* subgroups/species, i.e., *L. gasseri* subgroup, *L. ruminis* subgroup, *L. casei* subgroup, *L. reuteri* subgroup, *L. sakei* subgroup, *L. plantarum* subgroup, and *L. brevis* were detected in the samples from vaginally-born group, whereas only two members, i.e., *L. gasseri* subgroup and *L. brevis* were detected in the cesarean group. These data corroborate that several bacterial clades may already be present before birth in term infants’ gut. Further, lower detection rate of lactobacilli in cesarean-born babies suggests that the primary source of lactobacilli in infant gut is mainly from maternal vaginal and–to a lesser extent–anal microbiota during vaginal delivery, and that the colonization by some important *Lactobacillus* species is delayed in babies delivered via cesarean-section.

## Introduction

Gut microbiota plays a fundamental role in various aspects of health and numerous diseases, but the multifarious dynamics of early-life intestinal microbiota colonization are only now beginning to come to light. For decades, it had been believed that the fetus as well as the intrauterine environment is universally sterile, and the earliest microbial exposure and colonization of infant gut begins during delivery and promptly thereafter by the acquisition of maternal (vaginal, anal, and skin microbiota) and environmental bacteria. However, some recent studies have elegantly reported the presence of bacteria in placenta (Aagaard et al., 2014; Collado et al., 2016), amniotic fluid (Oh et al., 2010; Collado et al., 2016), and umbilical cord blood (Jimenez et al., 2005), thereby manifesting that the fetus is already exposed to a variety of bacteria *in utero*. Several studies have also detected various bacteria in the meconium of healthy babies (Gosalbes et al., 2013; Hu et al., 2013; Del Chierico et al., 2015; Hansen et al., 2015; Collado et al., 2016), further evidencing that the gut microbial colonization may already begin before birth. However, quantitative data on the types and particularly the population levels (i.e., the absolute count) of bacteria that may be present in the meconium is still sparse. Also, the information on how the composition of ‘1st-day flora’ differs between infants born vaginally or by cesarean section is limited. Given that the early-life microbiota plays important role in infant’s physiological, metabolic and immunological development (Matamoros et al., 2013; Arrieta et al., 2015; Kerr et al., 2015) and is strongly impacted by birth mode (Penders et al., 2006; Tsuji et al., 2012; Backhed et al., 2015; Nagpal and Yamashiro, 2015; Bokulich et al., 2016; Nuriel-Ohayon et al., 2016), the numeric data about the population levels of bacteria present in the first intestinal discharge of vaginally-and cesarean-born newborns could be very informative and relevant. In this milieu, we herein aimed to quantify the bacteria present in the meconium of healthy term infants born vaginally or by C-section. Bacteria were quantified by using a highly sensitive culture-independent reverse-transcription-quantitative-PCR (RT-qPCR) based analytical method that targets the bacterial 16S rRNA molecules (Matsuda et al., 2009). We applied this method because it provides quantitative data; whereas 16S rRNA gene-based sequencing methods are largely qualitative and do not provide numerical data of bacterial population levels. Moreover, it is highly sensitive, whereas DNA-targeted PCR methods have relatively lower sensitivity and hence may overlook several subdominant but important bacteria, especially in neonatal stages when the microbiota is underdeveloped. Another advantage is that it provides information on viable cells, whereas the results of DNA-based analyses might also include background DNA (e.g., free bacterial DNA, or DNA of dead bacteria) that may be present in prenatal niches such as amniotic fluid and placenta and also in meconium. We have previously validated that the counts obtained by this RT-qPCR method are equivalent to the bacterial counts enumerated by culturing and fluorescent *in situ* hybridization (FISH) methods and that the detection sensitivity of RT-qPCR is approximately 100- to 1000-fold higher than that of other molecular methods such as qPCR and terminal restriction fragment length polymorphism (t-RFLP) (Matsuda et al., 2007, 2009; Kubota et al., 2010; Kurakawa et al., 2015b). Therefore, owing to these advantages, we specifically implemented RT-qPCR for the present study. Herein we demonstrate the quantification of bacteria that are present in the first meconium of healthy term Japanese infants born vaginally or by C-section.

## Materials and Methods

### Subjects

The details of infants enrolled in this study are provided in **Table 1**. The study included 151 healthy full-term infants [134 vaginally-born (VG); 17 cesarean-born (CS)] whose mothers were recruited at the Gonohashi Obstetrics and Gynecology Hospital, Tokyo. The samples used in this study were part of a large cohort of Japanese infants enrolled in a longitudinal study monitoring the development of intestinal microbiota during the first 3 years of life, as reported in detail elsewhere (Tsuji et al., 2012). However, the meconium bacterial carriage was not previously investigated in detail, particularly in context to the delivery mode. In the present study, none of the babies were exposed to any type of formula-feed up to the point of first meconium; and all the infants as well as their mothers were apparently healthy with no indications of disease or intrauterine infection. All 17 cesarean cases were elective (planned) cesareans. The study design was approved by the ethical committee of Yakult Central Institute, Tokyo. Prior written informed consent was obtained from the parents or legal representatives of infants.

**Table 1:** General characteristics of the 151 babies enrolled in the study including birth mode, gender, sampling time, birth-weight and height, and antibiotic exposure.

	Vaginally-born	Cesarean-born
Number of infants	134	17
Male:Female	71:63	11:6
Sampling time (days after birth)	1 ± 0.3	1 ± 0.4
Birth-weight (kg)	2.98 ± 0.36	2.97 ± 0.32
Height (cm)	48 ± 1.8	48 ± 1.5
Antibiotics (birth to sampling)	3	0
Formula-feed (birth to sampling)	0	0


### Sample Collection

A spoonful (0.5–1.0 g) of the first intestinal discharge (obtained from the first diaper) was collected fresh in a fecal collection tube (Sarstedt AG & Co., Numbrecht, Germany) containing 2 ml RNA*later* (Ambion, Austin, TX, USA). Out of total 151 samples, 148 were passed within 24 h after birth whereas the rest three samples were discharged between 24 and 48 h. As described elsewhere (Tsuji et al., 2012), stool samples were also collected at age 3 and 7 days, 1, 3, and 6 months, and 3 years for follow-up analyses. All samples were collected at the hospital by following routine and standard clean techniques such as the use of sterile sample collection tube and spatula, and handler’s mouth masked, hands sanitized and gloved, and head capped while retrieving the sample. Immediately after collection, samples were stored in the refrigerator (3–4°C) anaerobically by using Anaero Pouch-Anaero (Mitsubishi Gas Chemical Company, Inc., Tokyo, Japan) and were sent immediately in a cooling box with refrigerants and anaero-packs to the research lab where these were stored at 3–4°C in a Biosafety Category II microbiology laboratory until further processing.

### Sample Processing, RNA Extraction and RT-qPCR

#### Primary Treatment

The samples were subjected to pretreatment according to the methods described previously (Matsuda et al., 2009). Briefly, the fecal samples were weighed and suspended in nine volumes of RNA*later* to make a fecal homogenate (100 mg feces/ml). In preparation for RNA extraction, 1ml of PBS(-) was added to 200 μl of fecal homogenate. The fecal homogenate was centrifuged at 4°C at 13,000 *g* for 10 min, the supernatant was discarded, and the precipitating pellet was stored at -80°C until RNA extraction.

#### RNA Extraction

RNA extraction was done by using previously described methods (Matsuda et al., 2009). Briefly, the thawed sample was resuspended in a solution containing 346.5 μl of RLT buffer (Qiagen Sciences, Germantown, MD, USA), 3.5 μl of β-mercaptoethanol (Sigma–Aldrich Co., St. Louis, MO, USA), and 100 μl of Tris-EDTA buffer. Glass beads (BioSpec Products, Inc., Bartlesville, OK, USA) (300 mg; diameter, 0.1 mm) were added to the suspension, and the mixture was subjected to a vigorous vortex procedure for 5 min using a ShakeMaster Auto apparatus (catalog no. BMS-A15; Bio Medical Science Inc., Tokyo, Japan). Acid phenol (Wako Pure Chemical Industries, Ltd., Osaka, Japan) (500 μl) was added, and the mixture was incubated for 10 min at 60°C. After phenol-chloroform purification and isopropanol precipitation, the nucleic acid fraction was suspended in 0.2 ml of nuclease-free water (Ambion, Inc.).

#### Reverse-Transcription-Quantitative-PCR (RT-qPCR)

Bacterial counts of various bacterial groups including total bacteria, *Clostridium coccoides* group, *C. leptum* subgroup, *Bacteroides fragilis* group, *Atopobium* cluster, *Prevotella*, *Bifidobacterium*, *Lactobacillus* subgroups and species, Enterobacteriaceae, *Enterococcus*, *Staphylococcus*, *Streptococcus*, *C. perfringens*, and *C. difficile* were analyzed by using a sensitive quantitative analytical system based on 16S or 23S rRNA molecules-targeted RT-qPCR, as per the methods described previously (Matsuda et al., 2009, 2012; Sakaguchi et al., 2010; Tsuji et al., 2012). Briefly, RT-qPCR was performed with a Qiagen OneStep RT-PCR kit (Qiagen GmbH, Hilden, Germany). Each reaction mixture (10 μl) was composed of 1X Qiagen OneStep RT-PCR buffer, 0.5X Q-solution buffer, each deoxynucleoside triphosphate at a concentration of 400 μM, a 1:100,000 dilution of SYBR green I (BioWhittaker Molecular Applications, Rockland, ME, USA), 0.4 μl of Qiagen OneStep RT-PCR enzyme mixture, and 5 μl of template RNA. Each primer set was added at a concentration of 0.6 μM. The reaction mixture was incubated at 50°C for 30 min for reverse transcription. The continuous amplification program consisted of one cycle at 95°C for 15 min, followed by 45 cycles at 94°C for 20 s, 55/60°C for 20 s, and 72°C for 50 s. The fluorescent products were detected in the last step of each cycle. A melting curve analysis was performed after amplification to distinguish the targeted PCR products from the non-targeted ones. The melting curve was obtained by slow heating at temperatures from 60 to 95°C at a rate of 0.2°C/s with continuous fluorescence collection. Amplification and detection were performed in 384-well optical plates with an ABI PRISM^®^ 7900HT sequence detection system (Applied Biosystems, Foster, CA, USA). Standard curves for the corresponding standard bacterial strains were generated by using *C*_q_ (quantification cycle) values and the corresponding cell counts, which were determined microscopically with the DAPI (4′,6-diamidino-2-phenylindole) staining method as previously described (Matsuda et al., 2009). To determine the target bacterial populations in the fecal samples, 1/20,000, 1/200,000, and 1/2,000,000 portions of the RNA solution were subjected to RT-qPCR. The *C*_q_-values in the linear range of the assay were applied to the analytical curve generated in the same experiment to obtain the corresponding bacterial count in each nucleic acid sample; this count was then converted to the count per sample. The details of analytical validation of specificities and sensitivities of these assays have been reported elsewhere (Matsuda et al., 2009, 2012; Sakaguchi et al., 2010). Briefly, the specificity of each primer set was confirmed against the total RNA fractions extracted from 10^5^ cells of the corresponding standard bacterial strain (Matsuda et al., 2009) by using RT-qPCR. The amplified signal was considered positive (+) at >10^4^ standard cells, positive/negative (±) at 10^4^ to 100 standard cells, and negative (-) at <100 standard cells. The amplified signal was also defined as negative (-) when the corresponding melting curve had a peak different from that of the standard strain. To determine the detection sensitivity, RNA fractions were extracted from culture samples of corresponding reference strain in the early stationary phase (24 h), and bacterial counts were determined microscopically by DAPI staining. Serial RNA dilutions corresponding to bacterial counts ranging from 10^-3^ to 10^5^ cells were assessed by RT-qPCR assay. The range of RNA concentrations at which there was linearity with *C*_q_-value was confirmed (*R*^2^ > 0.99). The details of the 16S or 23S rRNA gene targeted primers and the corresponding primer sequences, annealing temperatures and the minimum detection limits have been provided in the **Supplementary Table S2**.

### Statistical Analyses

The results of bacterial count (log_10_ cells per gram of feces) are expressed as mean ± standard deviation. The detection rate was expressed as the percentage of infants in which the specific bacterium was detected. During analysis in the spread-sheets to calculate the mean bacterial count and the detection rate, the cells were left blank if the specific bacterium was not detected (i.e., below the detection limit) in a sample. The count of genus *Lactobacillus* was expressed as the sum of the counts of six subgroups (*L. casei* subgroup, *L. gasseri* subgroup, *L. plantarum* subgroup, *L. reuteri* subgroup, *L. ruminis* subgroup, and *L. sakei* subgroup) and two species (*L. brevis* and *L. fermentum*). The comparisons of fecal bacterial counts by the mode of delivery were calculated by unpaired Student’s *t*-test. Differences in the detection rate of bacteria were calculated by Fisher’s exact probability test. *P* < 0.05 was considered statistically significant.

## Results

We detected several bacterial groups in the first meconium samples of both VG and CS infants. There was no significant difference in the birth weight, height or other generic parameters according to the birth mode (**Table 1**). The count and detection rate of intestinal bacteria are presented in **Table 2**. Overall, the meconium of 95% infants were found to harbor one or more types of bacteria; whereas the meconium of remaining 5% infants appeared to be sterile, i.e., no bacteria were detected in these samples. *B. fragilis* group, Enterobacteriaceae, *Enterococcus, Streptococcus*, and *Staphylococcus* were most prevalent members (detected in more than 50% infants), with counts ranging from 10^5^ to 10^8^ cells/g sample, followed by bifidobacteria, lactobacilli, *C. coccoides* group, *C. leptum* subgroup, *C. perfringens*, *Atopobium* cluster, and *Prevotella* (**Table 2**). Of total 151 infants, *C. difficile* was detected only in one baby (in VG group).

**Table 2:** Count and detection rate of major bacterial groups in the meconium of vaginally- and cesarean-born babies.

	Vaginally-born *n* = 134		Cesarean-born *n* = 17	
		
	Count^1^	Detection rate^2^	Count^1^	Detection rate^2^
Total bacteria^§^	6.8 ± 2.0	96.3	7.0 ± 1.9	88.2
Obligate anaerobes:				
*Clostridium coccoides* group	4.8 ± 1.6	20.1	4.8 ± 0.5	17.6
*Clostridium leptum* subgroup	4.8 ± 1.1	9.0	4.1 ± 0.3	23.5
*Bacteroides fragilis* group	5.3 ± 2.0	56.7	5.0 ± 2.3	52.9
*Prevotella*	5.2	1.5	ND	ND
*Bifidobacterium*	5.7 ± 1.7	32.1	5.7 ± 1.6	47.1
*Atopobium* cluster	6.0 ± 1.2	3.7	4.8	5.9
*Clostridium perfringens*	2.9 ± 0.9	11.2	2.8 ± 1.2	11.8
*Clostridium difficile*	3.8	0.7	ND	ND
Facultative anaerobes:				
Enterobacteriaceae	7.0 ± 1.8	53.7	5.9 ± 1.6	58.8
*Enterococcus*	5.0 ± 1.8	56.0	4.9 ± 1.8	64.7
*Staphylococcus*	5.4 ± 1.5	61.9	5.9 ± 1.7	41.2
*Streptococcus*	4.4 ± 1.2	52.2	3.9 ± 0.6	52.9
*Lactobacillus*^#^	3.7 ± 0.9	36.6	5.0	5.9^∗^


*^1^Mean ± SD of Log_10_ cells/g feces.*

*^2^Detection rate (%) was expressed as the percentage of infants in which the specific bacterium was detected.*

*^§^ Total bacteria are expressed as the sum of all the other bacteria listed in the table.*

*^#^The count of genus *Lactobacillus* was expressed as the sum of the counts of six subgroups (*L. casei* subgroup, *L. gasseri* subgroup, *L. plantarum* subgroup, *L. reuteri* subgroup, *L. ruminis* subgroup, and *L. sakei* subgroup) and two species (*L. brevis*, *L. fermentum*).*

*^∗^*P* = 0.012, vs. vaginally-born (Fisher’s exact probability test).*

*ND, not detected.*

**FIGURE 1:**
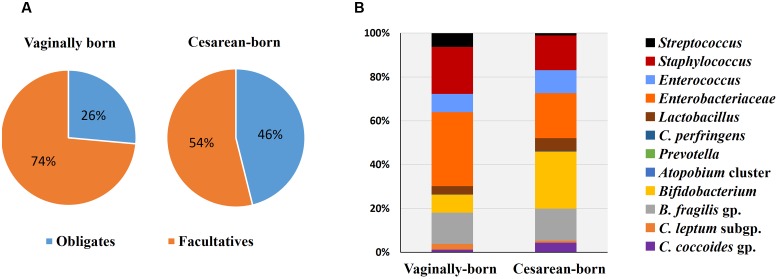
**Comparison of proportional ratio of obligatory anaerobic vs. facultative anaerobic bacteria** **(A)**, and relative proportions of different gut bacteria **(B)** in the meconium samples of vaginally- vs. cesarean-born infants. Proportions were calculated by using the original arithmetical number of the bacterial count and are expressed as the percent of numerical value of the total bacterial count. Obligates: *Clostridium coccoides* group, *C. leptum* subgroup, *C. perfringens*, *Bacteroides fragilis* group, and *Bifidobacterium*. Facultatives: *Lactobacillus*, Enterobacteriacea, *Enterococcus*, *Staphylococcus*, and *Streptococcus*.

**FIGURE 2:**
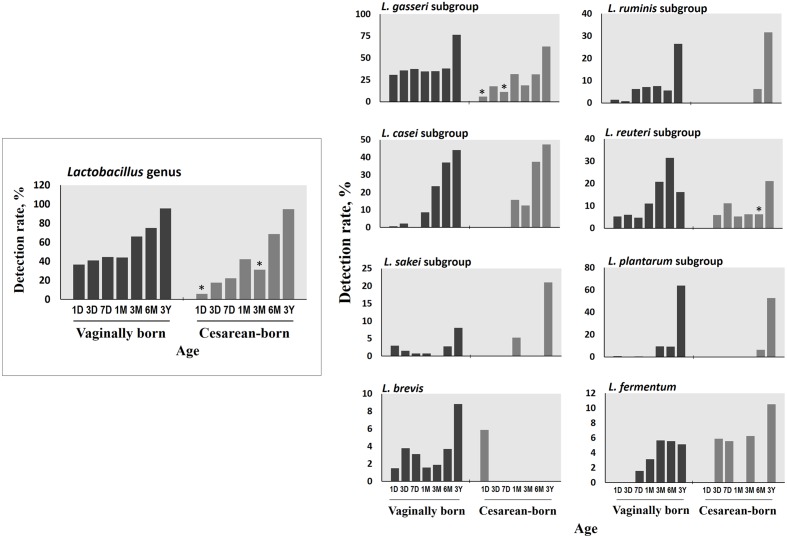
**Differences in the detection rate of *Lactobacillus* genus, subgroups and species between vaginally- and cesarean-born babies at different time-points during the first 3 years of life.** Detection rate (%) was expressed as the percentage of infants in which the specific bacterium was detected. The count of genus *Lactobacillus* was expressed as the sum of the counts of six subgroups (*L. casei* subgroup, *L. gasseri* subgroup, *L. plantarum* subgroup, *L. reuteri* subgroup, *L. ruminis* subgroup, and *L. sakei* subgroup) and two species (*L. brevis*, *L. fermentum*). ^∗^*P* < 0.05 vs. vaginally-born at same-point (Fisher’s exact test). Age (*x*-axis): 1, 3, and 7 days, 1, 3, and 6 months, 3 years.

Compared to VG infants, the meconium of CS infants were significantly less often colonized with *Lactobacillus* genus (6% vs. 37%; *P* = 0.01) (**Table 2**). Further examination of eight *Lactobacillus* subgroups and species revealed that *L. gasseri* subgroup was the most prevalent subgroup in VG infants (detected in 31% babies) whereas rest of the subgroups and species were detected only in less than 6% babies (**Table 3**). The detection rate of *L. gasseri* subgroup was significantly lower in CS babies compared to those born vaginally (6% vs. 31%; *P* = 0.04). Overall, total seven *Lactobacillus* subgroups/species, i.e., *L. gasseri* subgroup, *L. ruminis* subgroup, *L. casei* subgroup, *L. reuteri* subgroup, *L. sakei* subgroup, *L. plantarum* subgroup, and *L. brevis* were detected in the meconium samples of VG group, whereas only two members, i.e., *L. gasseri* subgroup and *L. brevis* were detected in CS group (*P* = 0.04). Other than lactobacilli, no significant difference was observed in the carriage of any other bacterial group in the meconium samples. However, the count of *Enterobacteriacea* was insignificantly lower in CS babies compared to VG babies (*P* = 0.058) (**Table 2**). Also, the overall ratio of facultative vs. obligate anaerobes (in proportion to total bacterial count) was significantly lower in CS babies than those delivered vaginally (*P* = 0.005) (**Figure 1A**); and accordingly, the overall proportional bacterial composition appeared to be slightly different in VG and CS babies (**Figure 1B**). Follow-up analysis of lactobacilli revealed that, compared to VG infants, the detection rate of *Lactobacillus* genus and several subgroups and species remained significantly or insignificantly lower in CS infants at different time-points during the first 6 months of life (**Figure 2**); but these differences tended to disappear by age 3 years.

**Table 3:** Count and detection rate of *Lactobacillus* subgroups and species in the meconium of vaginally- and cesarean-born babies.

	Vaginally-born *n* = 134		Cesarean-born *n* = 17	
		
	Count^1^	Detection rate^2^	Count^1^	Detection rate^2^
*L. gasseri* subgroup	3.6 ± 0.9	30.6	5.0	5.9^∗^
*L. ruminis* subgroup	2.9	1.5	ND	ND
*L. casei* subgroup	3.6	0.7	ND	ND
*L. reuteri* subgroup	3.1 ± 0.6	5.2	ND	ND
*L. sakei* subgroup	4.6 ± 0.6	3.0	ND	ND
*L. plantarum* subgroup	4.5	0.7	ND	ND
*L. brevis*	3.1	1.5	3.2	5.9
*L. fermentum*	ND	ND	ND	ND


*^1^Mean ± SD of Log_10_ cells/g feces.*

*^2^Detection rate (%) was expressed as the percentage of infants in which the specific bacterium was detected.*

*^∗^*P* = 0.04, vs. vaginally-born (Fisher’s exact probability test).*

*ND, not detected.*

## Discussion

To our knowledge, this is the largest birth cohort studied demonstrating a quantitative profile of the meconium bacterial microbiota in babies delivered vaginally or via C-section. Several remarkable studies have recently reported the presence of bacteria not only in meconium but also in placenta, amnion, and umbilical cord blood (Gosalbes et al., 2013; Hu et al., 2013; Del Chierico et al., 2015; Collado et al., 2016); but these were primarily based on DNA-based sequencing methods which, while being elegantly elaborative, may overlook several subdominant bacteria (e.g., ≤10^4^ cells/g feces), particularly during the earliest phase of life when the microbiota is immature. In this context, our data should fortify these evidences with important information of population levels of not only the predominant but also the subdominant bacteria that may inhabit the infant gut during critical developmental stages. We detected bacteria in the meconium of 95% babies while rest 5% samples appeared to be sterile (i.e., no bacteria detected). However, previous studies on term infants reported the presence of bacteria in 66% (using 16S rRNA gene-targeted FISH method) (Hansen et al., 2015) and 77% (using 16S/23S rRNA gene-targeted qPCR) (Martin et al., 2016) of total babies studied. This difference can most possibly be ascribed to differences in the detection sensitivities of the methods used, and hence could corroborate the advantage of high sensitivity of our RT-qPCR assays. We detected several bacterial clades, *albeit* with varying detection rates and numbers, in the meconium of both VG and CS infants (**Tables 2** and **3**), which clearly hint that the intrauterine environment harbors several bacterial communities which may provide the early inoculum for intestinal microbiota colonization. Further, these data show that the facultative anaerobes thrive–and hence are the first settlers–in the primitive gut, thereby pointing toward a relatively aerobic intestinal environment (Backhed et al., 2015; Del Chierico et al., 2015; Bokulich et al., 2016; Collado et al., 2016; Martin et al., 2016). This also suggests that the pioneer microbiota core is comprised predominantly of facultative anaerobes such as Proteobacteria, Staphylococci, Streptococci, Enterococci etc.; whereas obligates belonging to Firmicutes, Bacteroidetes, and Actinobacteria take over the predominance later on during infancy and early childhood, as also evident from the follow-up data of this cohort (**Supplementary Table S1**). This also concurs well with a recent study by our colleagues who reported that the 1st-day microbiota of healthy infants is by and large predominated by Enterobacteria or Staphylococci, but this predominance is mostly, rapidly transitioned to *Bifidobacterium*-dominated microbiota within a few days after birth (Matsuki et al., 2016).

Early-life microbiota colonization is strongly impacted by birth mode (Penders et al., 2006). During vaginal delivery, the infant is seeded with certain bacteria from the birth canal; but C-section bypasses this seeding thereby resulting in a different spectrum of pioneering microbiota (Dominguez-Bello et al., 2010; Backhed et al., 2015; Bokulich et al., 2016; Nuriel-Ohayon et al., 2016). Therefore, to determine whether–and how prominently–these differences exist in the first-pass samples, we compared the meconium microbiota of vaginally- vs. cesarean-born infants. The lower detection rate of *Lactobacillus* genus and the *L. gasseri* subgroup in the meconium of CS babies was intriguing. To know how long this difference persisted, we followed up the *Lactobacillus* carriage, and interestingly–*albeit* not surprisingly–VG and CS infants were found to exhibit dissimilar degrees of *Lactobacillus* detection rate at several time-points during the first 6 months of life, with detection rate being relatively lower in CS infants (**Figure 2**). It might also be possible that some vaginal materials are also transmitted from mother to baby during vaginal delivery that may have promoted the growth and activity of lactobacilli in vaginally-born babies; however, we were not able to verify this issue because of the unavailability of the maternal samples in this study. Interestingly, by using same RT-qPCR assays, we recently reported that the vaginal microbiota of healthy Japanese women of reproductive age is predominated numerically by *Lactobacillus* communities among which *L. gasseri* subgroup is a major constituent (Kurakawa et al., 2015a). Also, lactobacilli are rarely detected in amniotic fluid and placenta (DiGiulio et al., 2010; Aagaard et al., 2014). Collectively, these studies suggest a prominent mother-to-baby transmission of vaginal *Lactobacillus* communities during vaginal delivery, while indicating a delayed sampling of lactobacilli in CS babies’ gut. However, given the limitation of large difference between the numbers of vaginally- and cesarean-born infants, this finding needs to be corroborated in studies with comparable number of subjects in the two groups. Also, given that *L. gasseri* subgroup comprises several beneficial commensal species that can elicit probiotic health benefits such as anti-pathogenic activity, bacteriocin production, immune-modulation, and maintenance of gut homeostasis (Selle and Klaenhammer, 2013); studies are needed to investigate the impact, if any, of its lower/delayed colonization in CS babies. As for the clinical milieu, this delayed sampling does not seem to directly indicate any disease risk, because all these infants were apparently healthy (at least until age 3 years). Nevertheless, differences in *Lactobacillus* carriage in the ‘first flora’ justify further research and should be an exciting theme for future studies about viable therapeutic targets (probiotics, prebiotics, etc.) in context to the gut dysbiosis, particularly in cesarean-born and/ or preterm babies.

While the birth mode did not have a significant effect on the carriage of other bacteria (apart from lactobacilli), we noticed differences in the ratio of facultative vs. obligatory anaerobes and also in the relative proportions of different bacteria between VG and CS infants (**Figure 1**). Assuming VG babies as healthy controls, this might contemplate some sort of imbalanced microbiota in CS babies (Arboleya et al., 2012) and might also hint that the trajectory of C-section-associated gut dysbiosis may already instigate as early as the 1st day of life. Slightly higher count of facultative anaerobe community belonging to Enterobacteriaceae in VG babies might indicate the transmission of maternal perianal bacteria during birth, because enterobacteria are typical fecal bacteria and defecation during parturition is not uncommon (de Muinck et al., 2011). We have previously reported that the carriage of *B. fragilis* group, bifidobacteria and several other bacteria remain inconsistently lower in CS babies until age 6 months, but these differences begin to appear after 3–7 days of age (Tsuji et al., 2012). Together, these reports suggest that while the infant microbiota is definitely, strongly influenced by delivery mode, these differences (with the exception of lactobacilli) are not noticeable at meconium stage and become prominent thereafter (Backhed et al., 2015; Bokulich et al., 2016). This is perplexing and calls for further research, because if the initial dose and pool of microbes in VG and CS babies is similar, then what is it that drives the salient differences in the carriage of certain bacteria (such as *B. fragilis* group, bifidobacteria etc.) during subsequent stages. Nevertheless, our data show that irrespective of birth mode, the prenatal microbiome is characterized by specific founding bacterial clades which subsequently become more diverse with age as exposure to various environmental and dietary factors increases (**Figure 2**; **Supplementary Table S1**). While the source(s) of these bacteria in the meconium remain largely unclear, they are speculated to be of intrauterine origin and suggestive of swallowed amniotic fluid (Ardissone et al., 2014; Collado et al., 2016). Given that the meconium bacterial composition could reveal several features of maternal health (Hu et al., 2013) and might also be associated with several infantile diseases (Gosalbes et al., 2013), further research on the characteristics of these bacteria and their interaction with the host may lead to novel interventions and therapies for newborn’s health.

Our study did have some limitations most important of which was our inability to evaluate the influence of prenatal factors such as maternal diet, antibiotics, stress etc. because of the paucity of information. Also, the influence of maternal microbiota (e.g., fecal, vaginal, and skin microbiota) was not studied because maternal samples were not collected in this open observational study. Even though all samples were from first stools, the time of sampling may have varied in each case and hence might have some influence on the bacterial composition in babies who passed their first stool at an earlier vs. later time. However, because 148 out of total 151 samples were collected within the first 24 h of life, it is highly implausible that sampling time could have any significant bias on the results. While the possibility of environmental contamination during sample collection cannot be completely ruled out; we followed all standard and potential laboratory procedures to avoid as much as possible the chances, if any, of contamination. Moreover, the finding that 5% samples were found to be sterile (no detectable bacteria) also corroborates the sterility during sample collection. While no baby received any kind of formula-feed up to the point of first defecation, some of the babies might have been breast-fed during this short period. However, because of lack of precise information, we could not distinguish such infants to exclude the possibility of contamination with breast-milk’s bacteria. Nevertheless, given that all the mothers and babies were apparently healthy, the data could be extrapolated to the general population.

## Conclusion

In summary, our data demonstrate the quantitative picture of bacterial pool dwelling in neonate’s first meconium, and corroborate the evidence that the colonization of gut microbiota may begin already before birth, i.e., *in utero*. Furthermore, delayed/lower colonization of lactobacilli and relatively different proportions of facultative vs. obligatory anaerobes in the meconium of CS babies indicate that the elements of gut dysbiosis associated with birth mode may start building up as early as the 1st day of life. Given that early-life microbiota acquisition is crucial for newborn’s growth and development, our data underscore the need for broader studies to elucidate the sources, routes and significance of these microbial clades in the prenatal niches, as well as to investigate the impact of these primitive microbial exposures on newborn’s long-term health.

## Author Contributions

RN, HT, KN, and YY: conception and design of study. RN and HT: performed experiments. HT and KK: coordinated sample collection. RN and HT: analyzed data. RN, HT, KN, and YY: interpreted data. RN: drafted manuscript. RN, HT, TT, KN, KK, SN, and YY: edited and revised manuscript. RN, HT, TT, KN, KK, SN, and YY: approved final version of manuscript.

## Conflict of Interest Statement

RN is supported by postdoctoral research fellowship from Juntendo University. HT, TT, and KN are employees of Yakult Honsha Co. Ltd, Japan. SN and YY have received unrestricted research grants from Yakult Honsha Co. Ltd. The author KK declares that the research was conducted in the absence of any commercial or financial relationships that could be construed as a potential conflict of interest.
