# Supplementary material for: Sensitive Quantitative Analysis of the Meconium Bacterial Microbiota in Healthy Term Infants Born Vaginally or by Cesarean Section
Source: Front Microbiol. 2016 Dec 15;7:1997. doi: 10.3389/fmicb.2016.01997 (PMC5156933; doi:10.3389/fmicb.2016.01997)
Supplement: Supplementary file 1 [file Table_1.DOCX]

**Suppl. Table S1.** Count and detection rate of major bacterial groups in the feces of vaginally- and cesarean-born infants at 6 months and 3 years of age.

|  | Age 6 months | | | | Age 3 years | | | |
| --- | --- | --- | --- | --- | --- | --- | --- | --- |
|  | Vaginally-born | | Cesarean-born | | Vaginally-born | | Cesarean-born | |
|  | n=108 | | n=16 | | n=136 | | n=19 | |
|  | Count^1^ | Detection rate^2^ | Count | Detection rate | Count | Detection rate | Count | Detection rate |
| Total bacteria^§^ | 10.9 ± 0.4 | 100 | 10.9 ± 0.5 | 100 | 10.8 ± 0.3 | 100 | 10.8 ± 0.4 | 100 |
| *Clostridium coccoides* group | 7.6 ± 2.5 | 94 | 8.1 ± 2.6 | 88 | 9.9 ± 0.4 | 100 | 9.9 ± 0.4 | 100 |
| *Clostridium leptum* subgroup | 7.6 ± 1.8 | 63 | 8.0 ± 0.8 | 69 | 10.1 ± 0.6 | 100 | 10.1 ± 0.5 | 100 |
| *Bacteroides fragilis* group | 8.8 ± 2.5 | 77 | 6.5 ± 2.6**^*^** | 50**^*^** | 9.9 ± 0.4 | 100 | 10.0 ± 0.5 | 100 |
| *Prevotella* | 5.3 ± 0.8 | 69 | 5.3 ± 0.7 | 81 | 6.3 ± 1.0 | 52 | 6.8 ± 1.3 | 53 |
| *Bifidobacterium* | 10.2 ± 1.5 | 98 | 10.5 ± 1.5 | 100 | 10.2 ± 0.7 | 100 | 9.9 ± 1.1 | 100 |
| *Atopobium* cluster | 8.1 ± 1.5 | 89 | 7.9 ± 1.0 | 88 | 9.2 ± 0.6 | 100 | 9.1 ± 0.6 | 100 |
| *Clostridium perfringens* | 5.8 ± 2.3 | 46 | 6.9 ± 1.9 | 75 | 5.2 ± 1.2 | 57 | 5.4 ± 1.7 | 68 |
| *Clostridium difficile* | 5.8 ± 2.3 | 42 | 5.5 ± 2.8 | 38 | 4.7 ± 1.5 | 15 | 6.6 ± 1.2 | 11 |
| Enterobacteriaceae | 9.1 ± 0.8 | 100 | 9.2 ± 0.6 | 100 | 7.7 ± 0.7 | 95 | 7.6 ± 0.6 | 89 |
| *Enterococcus* | 8.3 ± 1.1 | 98 | 8.3 ± 0.8 | 100 | 7.6 ± 0.7 | 98 | 7.4 ± 1.1 | 95 |
| *Staphylococcus* | 6.8 ± 0.8 | 100 | 7.1 ± 0.7 | 94 | 5.3 ± 0.8 | 82 | 5.2 ± 0.6 | 89 |
| *Streptococcus* | 8.2 ± 0.8 | 100 | 8.0 ± 1.0 | 100 | 8.7 ± 0.5 | 100 | 8.7 ± 0.5 | 100 |
| *Lactobacillus*^#^ | 5.9 ± 2.1 | 75 | 5.9 ± 2.2 | 69 | 6.0 ± 1.6 | 96 | 6.4 ± 1.2 | 95 |

^1^Bacterial count is expressed as mean ± SD of Log_10_ cells/ g feces.

^2^Detection rate (%) was expressed as the percentage of infants in which the specific bacterium was detected.

^§^Total bacteria are expressed as the sum of all the other bacteria listed in the table.

^#^The count of genus *Lactobacillus* was expressed as the sum of the counts of six subgroups (*L. casei* subgroup, *L. gasseri* subgroup, *L. plantarum* subgroup, *L. reuteri* subgroup, *L. ruminis* subgroup, *L. sakei* subgroup) and two species (*L. brevis*, *L. fermentum*).

**^*^***P*<0.05 vs. vaginally born at same age (Count: unpaired Student’s *t*-test; Prevalence: Fisher’s exact probability test).
