# Supplementary material for: Sensitive Quantitative Analysis of the Meconium Bacterial Microbiota in Healthy Term Infants Born Vaginally or by Cesarean Section
Source: Front Microbiol. 2016 Dec 15;7:1997. doi: 10.3389/fmicb.2016.01997 (PMC5156933; doi:10.3389/fmicb.2016.01997)
Supplement: Supplementary file 2 [file Table_2.docx]

**Suppl. Table S2.** List of targeted bacterial groups and the corresponding primer sequences, annealing temperature and minimum detection limit.

| Target | Primer^*^ | Sequence (5’-3’) | Annealing temperature (°C) | Minimum detection limit (log_10_ cells/g feces) | Reference^#^ |
| --- | --- | --- | --- | --- | --- |
| *Clostridium coccoides* group^1^ | g-Ccoc-F  g-Ccoc-R | AAATGACGGTACCTGACTAA CTTTGAGTTTCATTCTTGCGAA | 55 | 3 | [1] |
| *Clostridium leptum* subgroup^2^ | sg-Clept-F  sg-Clept-R3 | GCACAAGCAGTGGAGT CTTCCTCCGTTTTGTCAA | 55 | 3 | [1] |
| *Bacteroides fragilis* group^3^ | g-Bfra-F2  g-Bfra-R | AYAGCCTTTCGAAAGRAAGAT CCAGTATCAACTGCAATTTTA | 50 | 3 | [1] |
| *Prevotella* | g-Prevo-F  g-Prevo-R | CACRGTAAACGATGGATGCC GGTCGGGTTGCAGACC | 55 | 5 | [1] |
| *Bifidobacterium* | g-Bifid-F  g-Bifid-R | CTCCTGGAAACGGGTGG GGTGTTCTTCCCGATATCTACA | 55 | 4 | [1] |
| *Atopobium* cluster^4^ | c-Atopo-F  c-Atopo-R | GGGTTGAGAGACCGACC CGGRGCTTCTTCTGCAGG | 55 | 4 | [1] |
| *Clostridium perfringens* | s-Clper-F  ClPER-R | GGGGGTTTCAACACCTCC GCAAGGGATGTCAAGTGT | 60 | 2 | [1] |
| *Clostridium difficile* | Cd-lsu-F  Cd-lsu-R | GGGAGCTTCCCATACGGGTTG  TTGACTGCCTCAATGCTTGGGC | 60 | 2 | [2] |
| Enterobacteriaceae | En-lsu-3F  En-lsu-3’R | TGCCGTAACTTCGGGAGAAGGCA TCAAGGACCAGTGTTCAGTGTC | 60 | 4 | [1] |
| *Enterococcus* | g-Encoc-F  g-Encoc-R | ATCAGAGGGGGATAACACTT ACTCTCATCCTTGTTCTTCTC | 55 | 3 | [1] |
| *Staphylococcus* | g-Staph-F  g-Staph-R | TTTGGGCTACACACGTGCTACAATGGACAA  AACAACTTTATGGGATTTGCWTGA | 60 | 3 | [1] |
| *Streptococcus* | gStr-F  gStr-R | AGCTTAGAAGCAGCTATTCATTC  GGATACACCTTTCGGTCTCTC | 60 | 3 | [3] |
| *Lactobacillus gasseri* subgroup | sg-Lgas-F  sg-Lgas-R | GATGCATAGCCGAGTTGAGAGACTGAT TAAAGGCCAGTTACTACCTCTATCC | 60 | 2 | [1] |
| *Lactobacillus ruminis* subgroup | sg-Lrum-F  sg-Lrum-R | CACCGAATGCTTGCAYTCACC GCCGCGGGTCCATCCAAAA | 60 | 2 | [1] |
| *Lactobacillus casei* subgroup | sg-Lcas-F  sg-Lcas-R | ACCGCATGGTTCTTGGC CCGACAACAGTTACTCTGCC | 60 | 2 | [1] |
| *Lactobacillus reuteri* subgroup | sg-Lreu-F  sg-Lreu-R | GAACGCAYTGGCCCAA TCCATTGTGGCCGATCAGT | 60 | 2 | [1] |
| *Lactobacillus sakei* subgroup | sg-Lsak-F  sg-Lsak-R | CATAAAACCTAMCACCGCATGG TCAGTTACTATCAGATACRTTCTTCTC | 60 | 2 | [1] |
| *Lactobacillus plantarum* subgroup | sg-Lpla-F  sg-Lpla-R | CTCTGGTATTGATTGGTGCTTGCAT GTTCGCCACTCACTCAAATGTAAA | 60 | 2 | [1] |
| *Lactobacillus brevis* | s-Lbre-F  s-Lbre-R | ATTTTGTTTGAAAGGTGGCTTCGG ACCCTTGAACAGTTACTCTCAAAGG | 55 | 2 | [1] |
| *Lactobacillus fermentum* | LFer-1  LFer-2 | CCTGATTGATTTTGGTCGCCAAC ACGTATGAACAGTTACTCTCATACGT | 55 | 2 | [1] |

^1^*C. coccoides* group (Clostridial cluster XIVa) includes *C. symbiosum, C. asparagiforme, C. hathewayi, C. indolis, C. celerecrescens, C. sphenoides, C. nexile, C. scindens, C. hylemonae, Blautia coccoides, B. hydrogenotrophica, B. luti, B. schinkii, B. hansenii, B. producta, Eubacterium ventriosum, Eu. hallii, Eu. eligens, Eu. rectale, Eu. ramulus, Ruminococcus obeum, Rum. gnavus, Rum. torques, Rum. lactaris, Anaerostipes caccae, Roseburia intestinalis, Coprococcus eutactus, Coprococcus Comes, Dorea longicatena, Dorea formicigenerans,* and *Fusicatenibacter saccharivorans*.

^2^*C. leptum* subgroup (Clostridial cluster IV) includes *C. leptum, C. viride, C. cellulosi, C. sporosphaeroides, Eubacterium desmolans, Eu. siraeum, Faecalibacterium prausnitzii, Anaerofilum agile, Anaerofilum pentosovorans, Ruminococcus albus, Rum. callidus, Rum. flavefaciens,* and *Rum. bromii.*

^3^*B. fragilis* group includes *B. fragilis, B. vulgatus*, *B. ovatus*, *B. thetaiotaomicron, B. caccae, B. acidofaciens, B. stercoris, B. eggerthii, B. uniformis, B. heparinolyticus,* and *B. zoogleoformans*.

^4^*Atopobium* cluster includes *A. minutum, A. fossor, A. rimae, A. parvulum, Eggerthella lenta, Denitrobacterium detoxificans, Coriobacterium glomerans, Coriobacterium sp.EKS03, Collinsella aerofaciens, Collinsella intestinalis, Collinsella stercoris, Slackia exigua, Slackia heliotrinreducens,* and *Cryptobacterium curtum*.

^*^All the primers target 16S rRNA genes, except for Cd-lsu-F/R (*Clostridium difficle*), En-Isu-3F/En-Isu-3’R (Enterobacteriaceae) and g-Str-F/R (*Streptococcus*) which targets 23S rRNA genes.

^#^[1] Matsuda et al. 2009; [2] Matsuda et al. 2012; [3] Sakaguchi et al. 2010.
